# Low recovery of bacterial community after an extreme salinization-desalinization cycle

**DOI:** 10.1186/s12866-018-1333-2

**Published:** 2018-11-23

**Authors:** Yang Hu, Chengrong Bai, Jian Cai, Keqiang Shao, Xiangming Tang, Guang Gao

**Affiliations:** 1 State Key Laboratory of Lake Science and Environment, Nanjing Institute of Geography and Limnology, Chinese Academy of Sciences, 73 East Beijing Road, Nanjing, 210008 China; 2 University of Chinese Academy of Sciences, Beijing, 100000 China

**Keywords:** Bacterial community, Salinization-desalinization cycle, Recovery, Seed bank, Osmosensor capacity

## Abstract

**Background:**

Understanding the recovery of bacterial communities after extreme environmental disturbances offers key opportunities to investigate ecosystem resilience. However, it is not yet clear whether bacterial communities can rebound to their pre-disturbance levels. To shed light on this issue, we tracked the responses of bacterial communities during an extreme salinization-desalinization cycle.

**Results:**

Our results showed that salinization-up process induced an ecological succession, shifting from a community dominated by *Betaproteobacteria* to *Gammaproteobacteria*. Within the desalinization-down process, taxon-specific recovery trajectories varied profoundly, with only *Gammaproteobacteria* returning to their initial levels, of which *Alphaproteobacteria* was the most prominent member. The α-diversity indices gradually increased at oligosaline environment (0.03‰ to 3‰) and subsequently decreased profoundly at hypersaline condition (10‰ to 90‰). However, the indices did not return to pre-disturbance level along the previous trajectory observed during the desalinization. Approximately half of the original OTUs were not detected during desalinization, suggesting that the seed bank may be damaged by the hypersaline environment. Moreover, Phylogenetic Investigation of Communities by Reconstruction of Unobserved States (PICRUSt) implied that the osmosensors’ capacity of bacterial communities was also impaired by the hypersaline condition.

**Conclusions:**

These results suggested that the bacterial communities showed a low recovery after the extreme salinization-desalinization cycle.

**Electronic supplementary material:**

The online version of this article (10.1186/s12866-018-1333-2) contains supplementary material, which is available to authorized users.

## Background

Disturbance, defined as an event that triggers the environmental change, has lasting (positive or negative) influences on every level of biological organization at a broad range of spatio-temporal scales. Following from previous studies, it has been established that disturbance displaces or damages individuals to create opportunity for new individuals to establish themselves, leading to community succession [1, 2]. A series of concepts have been proposed to elucidate the community patterns associated with disturbance, including stability, resilience, and variability [3–5]. Inherently, disturbance is diverse with respect to either endogenous and exogenous or either natural and anthropogenic origins. Thus, the community responses to disturbance are also correspondingly diverse [6–8]. However, despite of these rich responses, little is known within the context of community robustness that whether community can recover to its pre-disturbance level after the disturbance [9–11].

Microbes are the major contributors for ecosystem functions, playing an essential role in organic matter mineralization, nutrient regeneration, and energy flow [12, 13]. Thus, the robustness of microbial communities to a disturbance is essential for understanding the ecosystem stability. On one hand, some ecological theories (e.g. niche theory and species-sorting process), which are characterized by deterministic mechanisms, potentially argue that the microbial community will eventually rebound to its initial level after a disturbance [14, 15]. For instance, aquatic bacterial communities generally matched their initial composition by the 10th day after lake mixing [11]. Similarly, bacterial diversity is also comparable with its initial levels on day 5 after a heavy *Microcystis* bloom [16]. These results suggest that there is only one fate for a bacterial community in a given environment. However, modern ecology has recently begun to challenge these dogmas. The failure or incomplete recovery of a bacterial community has been repeatedly recorded after specific disturbances, such as frequent antibiotic perturbation [10] and violent wind-disturbance [17]. Advocators challenging a single-fate model claim that the succession trajectory of a microbial community is not only controlled by deterministic mechanisms, but is also subjected to stochastic processes, such as dispersal limitation, priority effect, and ecological drift [18–21]. Thus it is expected that there would be multiple end-states for bacterial communities in a given environment [7, 22]. As a consequence, the microbial community may not rebound to its pre-disturbance level. Given these two contrary arguments, it is crucial to provide new insights into bacterial community recovery in order to better understanding the ecology of disturbance.

As one of the most influential factors, salinization is a heavy disturbance to bacterial communities and defines the distribution of a bacterial community in various habitats [23, 24]. At the individual level, salinization threatens the bacterial species with a drastic loss of water due to high osmotic pressure [25], leading to specific functions being largely inhibited, such as respiration [26] and nitrification [27]. At the community level, salinization induces an ecological succession, moving from a community dominated by halosensitive to halotolerant species [28–30]. For instance, *Betaproteobacteria*, the most prominent group in freshwater, is gradually displaced by *Gammaproteobacteria* along a saline gradient [31–34]. Notably, salinity is not always constant but fluctuates over time in natural ecosystems. In this case, the bacterial community is expected to not only suffer from salinization, but also from subsequent desalinization [35]. Although desalinization causes a symmetric change in osmotic pressure relative to salinization, there is a knowledge gap surrounding whether the bacterial community is able to recover its pre-disturbance level after desalinization. Therefore, the salinization-desalinization cycle provides a unique model for to understanding the robustness of bacterial communities.

To offer insights into the recovery of a microbial community, we performed a mesocosm experiment to investigate responses to the salinization-desalinization cycle. Specifically, the microbial community is manipulated to through six salinity levels from freshwater to extreme saline water (0.03‰, 1‰, 3‰, 10‰, 35‰, and 90‰), and then returning in reverse fashion to freshwater (90‰, 35‰, 10‰, 3‰, 1‰, and 0.03‰). The selection criteria for the salinity levels is based on natural salinity ranging from 0.03‰ (freshwater) to 35‰ (ocean water), and even up to 90‰ (hypersaline water) [36, 37]. Although there is an inverse relationship between disturbances frequency and magnitude (large disturbance seldom occurs, while small disturbance occurs frequently), disturbance of extreme salinity is inherently difficult to study in nature. However, the responses of a microbial community after extreme salinity are essential to understanding their recovery ecology [38, 39]. By investigating how bacterial community might responds to a salinization-desalinization cycle, we attempt to describe the microbial community’s ability to recover to its pre-disturbance levels.

## Results

### Physicochemical characteristics during the experiment

The major physicochemical characteristics of experimental water were tracked (Additional file 1: Table S1). In the control group, all environmental properties showed minor variation, indicating the steady state of the system during the experiment. Within the treatment group, only the salinity increased from freshwater (0.03‰) to the hypersaline condition (90‰), and then symmetrically recovered to freshwater (0.03‰) as described. Other major environmental factors showed no significant variation during the experimental period.

### The overall bacterial community diversity during salinization-desalinization cycle

We selected the universal forward primer 798F and reverse primer 1068R to amplify bacteria and archaea. The coverage of bacteria was 98.0% for both forward and reverse primer, while that of archaea was 96.6 and 99.2%, respectively (https://www.arb-silva.de/). However, the Illumina sequencing results showed that only 5 OTUs belonging to archaeal group were detected, which accounted for < 1% within the prokaryotic community. This finding suggested the archaeal community was particular rare relative to bacterial community. Based on the archaeal rarity, we only focused on the recovery of bacterial community during the salinization-desalinization cycle.

Our sequence-processing strategy yielded an average of 97,143 raw reads from each sample with a length of approximately 225 bp. After trimming, screening and removing chimeras, 67,309 high quality sequences for each sample were obtained, which were clustered into 2,418 OTUs across all 22 samples. Rarefaction curves of OTUs were used to estimate bacterial diversity among the salinization-desalinization cycle (Additional file 2: Figure S1). All rarefaction curves reached a plateau indicating that the sequencing depth has been guaranteed. Also, the high Good’s coverage (from 99.03 to 99.61%) supported the sufficient sequencing effort.

**Fig. 1:**
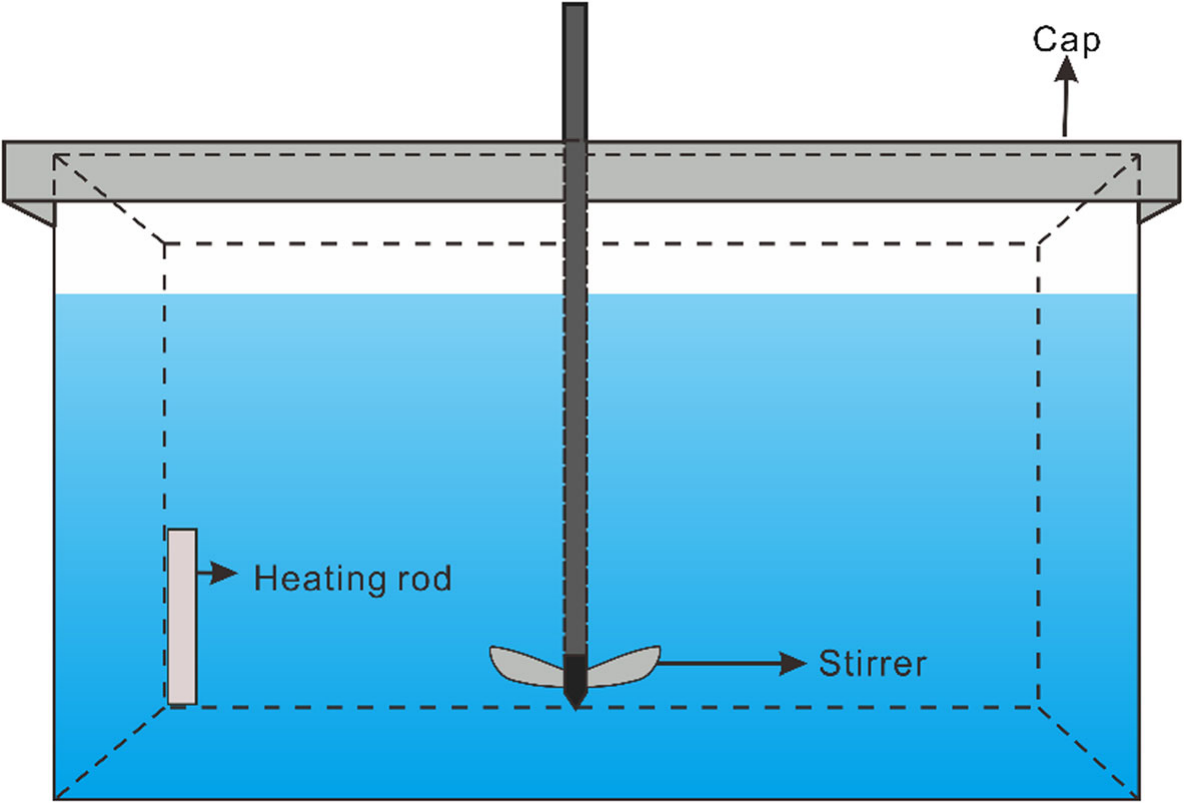
The α-diversity of bacterial communities of control and treatment groups based on Shannon index, Pielou index, Phylogenetic diversity and number of OTUs. The horizontal axis represents the status of salinity, S represents salinization, D represents desalinization, and the numbers after each letter represents the salinity in parts per thousand

**Fig. 2:**
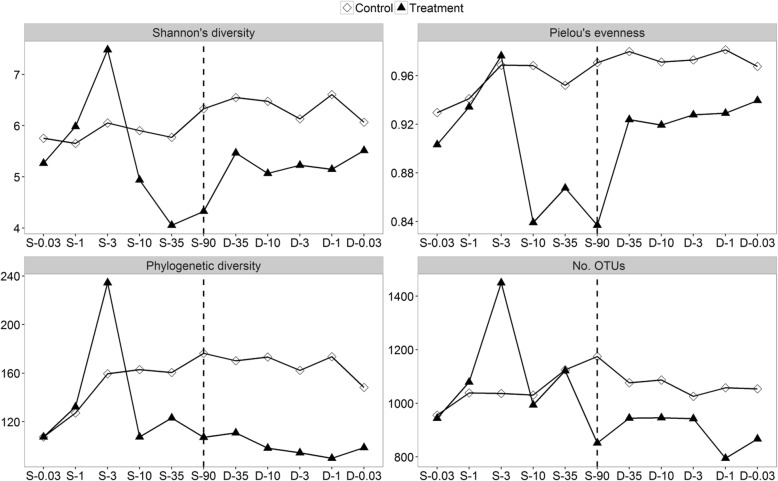
The persistence and appearance of bacterial species during the salinization-desalinization cycle. The horizontal axis represents the status of salinity, S represents salinization, D represents desalinization, and the numbers after each letter represents the salinity in parts per thousand

To characterize bacterial community robustness, we analyzed α-diversity patterns during the salinization-desalinization cycle (Fig. 1). In the control group, all diversity indices fluctuated and undulated slightly through whole experimental period. During the salinization-up process, our results showed two contrary trends of bacterial diversity. Specifically, all diversity indices increased at salinity from 0.03‰ to 3‰, whereas they drastically dropped at salinity from 10‰ to 90‰. Thus, there were two salinity spectrums for bacterial communities during the salinization process: oligosaline environment (from 0.03‰ to 3‰) and hypersaline environment (from 10‰ to 90‰). Within the subsequent desalinization-down process, although Shannon and Pielou indices showed an acute increase, values were still significantly lower than the control group (*t*-test, *P* < 0.001). Phylogenetic diversity and the number of OTUs were also not comparable with the control group (*t*-test, *P* < 0.001). Thus, diversity observations indicate that the bacterial communities did not recover to pre-disturbance levels after an extreme salinization-desalinization cycle. In order to explore the persistence, appearance and loss of OTUs within the salinization-desalinization cycle, we assigned OTUs that were detected in the control group to original OTUs and assigned OTUs that only appeared in the treatment group to unique OTUs (Fig. 2). The original OTUs continuously dropped steadily from 89.38 to 50.46% within salinization-up process and oscillated around 55.80% within the desalinization-down process. Consistently, the loss of OTUs increased gradually within the salinization and maintained a constant presence during desalinization. By contrast, there is an acute increase in the unique OTUs at salinity from 0.03‰ to 3‰.

**Fig. 3:**
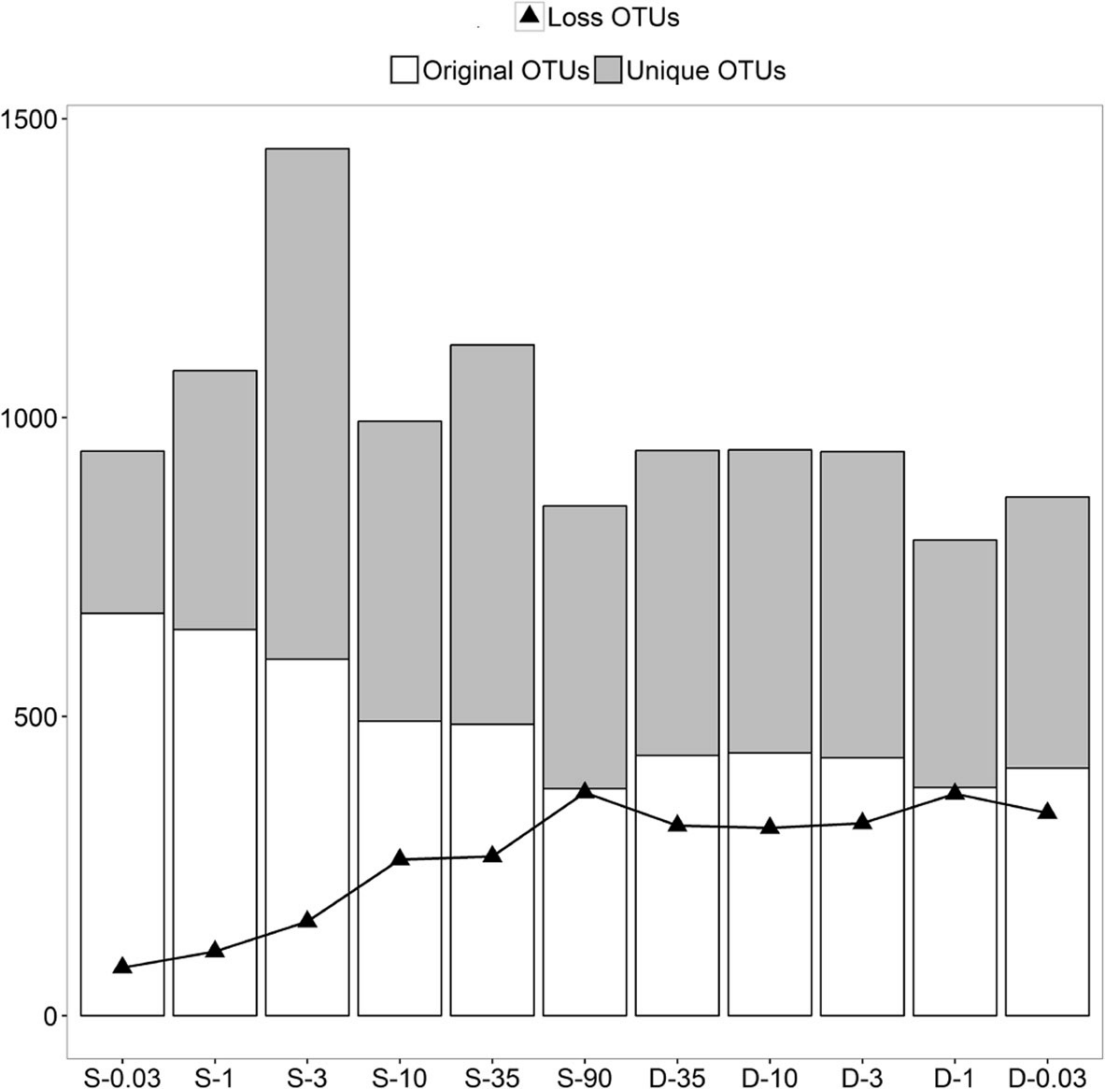
Non-metric multidimensional scaling analysis based on Bray-Curtis distance. The horizontal axis represents the status of salinity, S represents salinization, D represents desalinization, and the numbers after each letter represents the salinity in parts per thousand

We next assessed the β-diversity of bacterial communities during the salinization-desalinization cycle. With NMDS based on the Bray-Curtis similarities, salinization-up process clearly induced an ecological community succession, which deviated from that observed in the control group (Fig. 3). Analyses using the (un)weighted UniFrac method also provided similar results (Additional file 3: Figure S2). The PERMANOVA test also indicated that salinization communities were significantly different from the control group (*P* < 0.01). Of particular interest, salinization communities experienced an acute change between 3‰ and 10‰. And bacterial communities were significantly different between from 0.03‰ to 3‰ as well as from 10‰ to 90‰ (*P* < 0.05). This finding again implied that there were two salinity spectrums for bacterial communities: oligosaline and hypersaline environments. Strikingly, the desalinization communities did not rebound to pre-disturbance levels along their previous trajectories (Fig. 3). During the desalinization process, variability was evident, but was constrained around an average community composition that was generally stable. The communities were still visualized closer to the hypersaline community but significantly differed from their original control group (*P* < 0.01). Taken together, these observations may suggest an incomplete recovery of bacterial communities after an extreme salinization-desalinization cycle.

### Bacterial community profiles during the salinization-desalinization cycle

Within the control community, a total of 18 phyla were detected in all samples, with the top 3 accounting for approximately 86.5% of the whole community. Specifically, Proteobacteria (69.23%) was the most abundant phylum in all samples, followed by Bacteroidetes (12.31%) and Verrucomicrobia (11.28%). Further analysis at lower taxonomic level showed that prominent classes consisted of *Betaproteobacteria* (49.21%), *Alphaproteobacteria* (9.04%), and *Gammaproteobacteria* (3.24%). The presence of all phylotypes was fairly constant during the experimental period (Additional file 4: Figure S3).

**Fig. 4:**
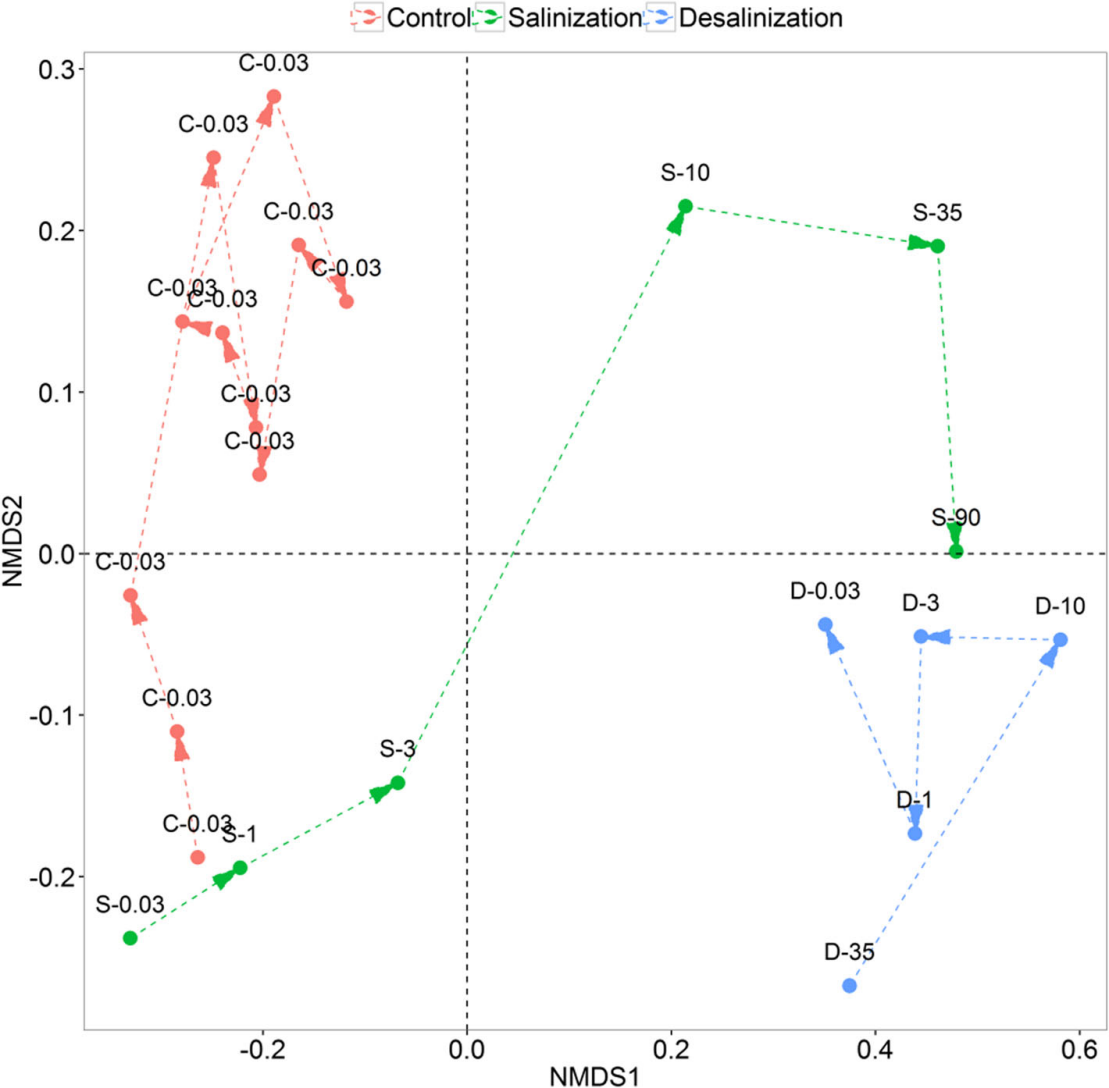
Phylum-level changes of bacterial community composition during salinization-desalinization cycle. The horizontal axis represents the status of salinity, S represents salinization, D represents desalinization, and the numbers after each letter represents the salinity in parts per thousand

There were distinct responses to the salinization-desalinization cycle among phylogenetically coherent groups at the class level (Fig. 4). During the salinization-up process, *Betaproteobacteria* maintained their dominance at salinity up to 1‰, accounting for 65.28%. However, their proportion drastically declined to 0.04% at salinity of 3‰, even decreased to 0.02% at the hypersaline environmental range (from 10‰ to 90‰). *Alphaproteobacteria* and *Planctomycetacia* both increased at the oligosaline condition. However, *Alphaproteobacteria* oscillated around 19.00% at the hypersaline environment, whereas *Planctomycetacia* largely declined to 1.64%. By contrast, *Gammaproteobacteria* continuously increased during the salinization and bloomed to become the most prominent group at salinity of 90‰.

Taxon-specific recovery trajectories varied markedly during the desalinization-down process. *Betaproteobacteria* increased from 4.40 to 12.55% with the decreasing salinity. However, their recovered proportion was still not comparable with their initial level. Instead, *Alphaproteobacteria* continued to increase to 50.42% as the most prominent member after the desalinization. Similarly, *Sphingobacteria* also exhibited an increase to around 19.26%, which was significantly higher than their original proportion (2.35%). Strikingly, only *Gammaproteobacteria* showed a symmetric pattern during the salinization-desalinization cycle, decreasing from 71.89 to 9.27%. Together, the shifts of these members implied that bacterial community composition did not recover to the initial level after the disturbance.

### Predictive metagenome analysis

**Fig. 5:**
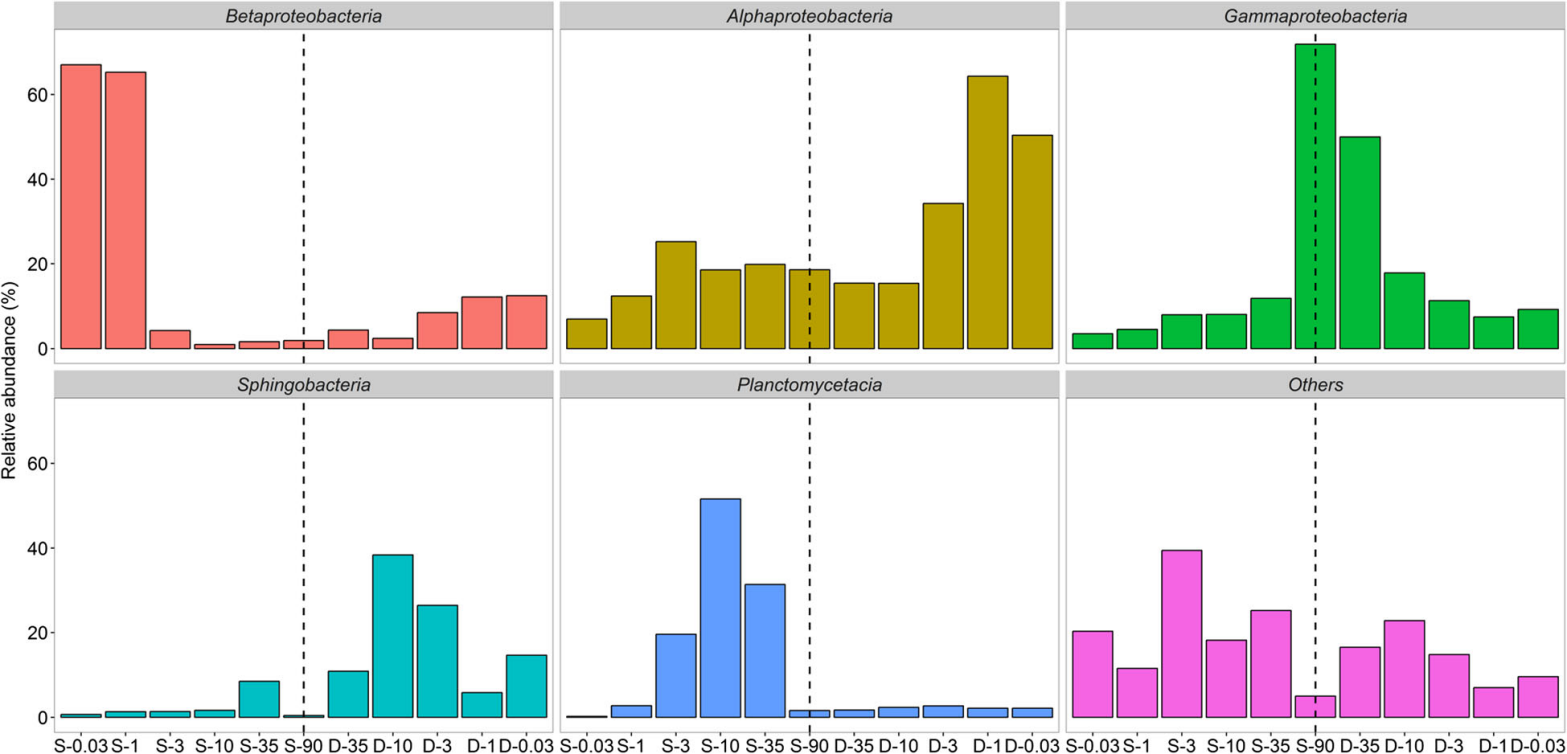
The abundance of five osmotic pressure regulation-regulated pathways

We used the PICRUSt program to predict metagenome functional content based on the KEGG classification. The nearest sequenced taxon index (NSTI) was employed to quantify the availability of nearby genome representatives for each sample (Additional file 5: Table S2). We mainly focused on five pathways (as defined by KEGG above) during the salinization-desalinization cycle: DNA repair and recombination proteins, DNA replication proteins, MAPK signaling pathway-yeast, inorganic ion transport, and two-component system (Fig. 5). All these pathways are involved in osmotic regulation. There were no significant differences in these pathways between the control groups and the oligosaline condition (all *P* > 0.05). As the salinity continuously increased to hypersaline condition they were all significantly depleted from the oligosaline to hypersaline environment (all *P* < 0.05). However, they did not clearly increase as the salinity decreased (all *P* > 0.05). None of these five osmotic regulation pathways rebounded to their previous levels in freshwater controls.

## Discussion

Over the last few decades, significant progress has been achieved in bacterial community ecology, however, characterizations of their recovery from disturbance are still largely unexplored. This is mainly because few studies follow bacterial communities over the time-course, but instead focus on the community sensitivity or immediate responses to disturbance [11, 40]. To address whether the bacterial community can recover to its initial state after a large disturbance, the current study artificially manipulated the bacterial community to through an extreme salinization-desalinization cycle. Our results showed that salinization-up process strongly induced an ecological succession in the bacterial community, however, the community did not recover to its pre-disturbance level along the previous trajectory during desalinization-down process.

The results of this study highlight that the bacterial community became richer within the increasing salinity range from 0.03‰ to 3‰. As has been stated above, salinity generally threatens the cell with a drastic loss of water due to higher osmotic pressure. Thus, it is widely held that there is a decrease in diversity for biota, particularly for floral and faunal species [41, 42]. However, this pattern seems to be less reproducible for bacterial communities. Relevant work has recorded that the bacterial taxon richness increased with increasing salinity up to a value of 1‰ [36]. A similar study also observed no significant decrease in bacterial diversity until salinity was 6.86‰ [31]. As the typical freshwater group, *Betaproteobacteria* maintained their dominance at salinity from 0.03‰ to 3‰, with a relative abundance of 66.15%. Concurrently, *Alphaproteobacteria* and *Gammaproteobacteria*, which are represented in saltwater habitats, clearly increased in the oligosaline condition. Therefore, the salinity-diversity pattern of bacterial communities may be related to the greater niche availability for both halotolerant and halosensitive bacteria at a moderate salinity level [34, 36].

Subsequent to experiencing oligosaline conditions, bacterial community diversity declined sharply at hypersaline environment from 10‰ to 90‰, which is in accordance with previous studies [28, 43]. This finding suggests that hypersaline conditions pose ecological barriers to the bacterial communities. Although the vast majority of bacteria have evolved mechanisms to cope with increasing osmolarity, some species will extinct as extracellular osmotic pressure exceeds their tolerance [25]. This is inferred from an acute of loss OTUs during the hypersaline environment. Indeed, according to Wright’s species-energy theory, a poorly productive environment leads to less diversity [37, 44, 45]. Previous work has documented that extreme saline environments potentially affect the physiological and biochemical functions of bacterial species [46], resulting in reduced the bacterial primary productivity [27, 43, 47]. As a consequence of this less robust environment, it is expectable that bacterial diversity dramatically decreased at the hypersaline conditions.

The current study details, importantly, that the bacterial communities did not recover to their previous levels after the desalinization-down process. Taxon-specific recovery trajectories varied profoundly, with only *Gammaproteobacteria* returning to their initial level (Fig 3). Also, the post-disturbance bacterial diversity was not comparable with its original state. Instead, a stable composition formed that was significantly different from the pre-disturbance state. This incomplete recovery is corroborated by previous experimental and observational evidence [10, 17]. From these key findings we attempt to propose two possible explanations. First, as a reservoir of dormant individuals that are capable of being resuscitated when environmental condition turns favorable, seed bank has been fundamentally used by a wide range of bacterial taxa [48, 49]. The importance of a seed bank for community recovery from disturbance via the storage effect has been theoretically and empirically demonstrated [50–52]. However, such storage is not unconditional: survival of individual cells in the seed bank is the prerequisite for community recovery [53]. As described above, the bacterial community were exposed to a wide salinity spectrum from 0.03‰ to 90‰. Actually, the salinity of most water never exceeds 35‰ [34, 54]. In this regard, most freshwater and oligosaline bacterial individuals may not be expected to survive after the extreme 90‰. This speculation was confirmed by the fact that almost half of the original OTUs were not detected from desalinization process. Consequently, there is no complete reservoir for the bacterial community to utilize for return to a full, pre-disturbance level. A second explanation is presented for recovery abilities of bacterial communities, namely that dormant organisms must be able to interpret the signals associated with a favorable environment, otherwise they will miss opportunities to resuscitate [49, 55, 56]. According to the PICRUSt, we found that five pathways, all of which operate as osmosensors in the transduction of osmotic pressure signals, were significantly inhibited by the extreme hypersaline condition and failed to recover during desalinization. Thus, it is assumed that the sensory capacity of bacterial communities may be impaired by a hypersaline environment. From this study, PICRUSt provides interesting caveats regarding its utility. For instance, this analytical method is only as good as the database of human systems, but it may not be adequate enough to describe that of environmental ecosystem [57]. As a consequence, more accurate approaches are required in future to provide greater evidence of signal interpretation in bacterial community recovery.

Many studies have suggested that bacterial communities are highly capable of recovery after specific disturbance, such as drying-wetting cycle, warming-cooling cycle and lake mixing [11, 38, 58, 59]. Thus, it is important to reconcile the two seemingly contradictory results. Relative to moderate perturbation, it is a familiar ecological phenomenon that extreme disturbance triggers regime shifts in communities: the return of external condition to their former state may not reverse such changes in community composition [60]. For instance, Dethlefsen and Relman found that the composition of the gut microbiota stabilized 2 months after the conclusion of their experienced disturbance, but there were significant alterations from its initial state [10]. Similarly, Lazarevic et al. observed a substantial but incomplete recovery of the salivary bacterial community even 3 weeks after antibiotic treatment [61]. We therefore find it necessary to highlight that any attempt to understand recovery of communities must be placed in the context of disturbance extent, duration and intensity [6, 62]. Additionally, we also note that variation in observed recovery patterns may be time-scale related. Woodward et al. found that most populations of a stream community returned to their pre-disturbance state in about 3 years, however, some took up to a decade to recover after extreme flood-drought cycle [9]. Thus, it is likely that intervals of 2 weeks’ time are too short for a complete recovery.

Our results of this study were obtained from a simplified experimental setup under laboratory condition, leading to several caveats which were gained for future considerations. Firstly, next generation sequencing cannot differentiate between viable and non-viable bacteria. Consequently, it is still unknow how culturable bacteria respond to the salinization-desalinization cycle. Secondly, the recovery of microbial community is not only influenced by abiotic factors, but also by biotic factors [63]. Specifically, microbes live in complicated networks through a multitude of interactions (e.g. competition, mutualism and antagonistism) [64], which govern the dynamics of microbial community recovery. However, current study did not reveal how these interactions guide the recovery strategy during the salinization-desalinization cycle. Finally, a set of bacterial communities are linked in natural ecosystem by dispersal of multiple species that are referred to as metacommunities [15]. In this case, the recovery of a bacterial community is controlled by inoculation of individual species from other habitats, such as sediment and air. [22]. The focus of the current research was a single habitat, namely water, so we cannot address the role of exogenous bacterial inoculation on community recovery. Collectively, we view these recognized limitations as an extremely exciting area for future work.

## Conclusions

This study provided insights into the recovery ecology of bacterial communities after an extreme disturbance. Current results showed that an oligosaline environment promotes diversity within the bacterial community, which may be related to a greater niche availability for both halotolerant and halosensitive bacteria at a moderate salinity level. However, a hypersaline environment poses ecological barriers to the bacterial communities, leading to their functional deficit and even annihilation. After desalinization-down process, the bacterial communities did not recover to their initial states. The possible underlying mechanism may be that the extreme hypersalinity impairs the seed bank of the bacterial community, as well as the capacity of their osmosensors to identify a return to favorable environmental conditions for greater recovery.

## Methods

### Experimental set-up

**Fig. 6:**
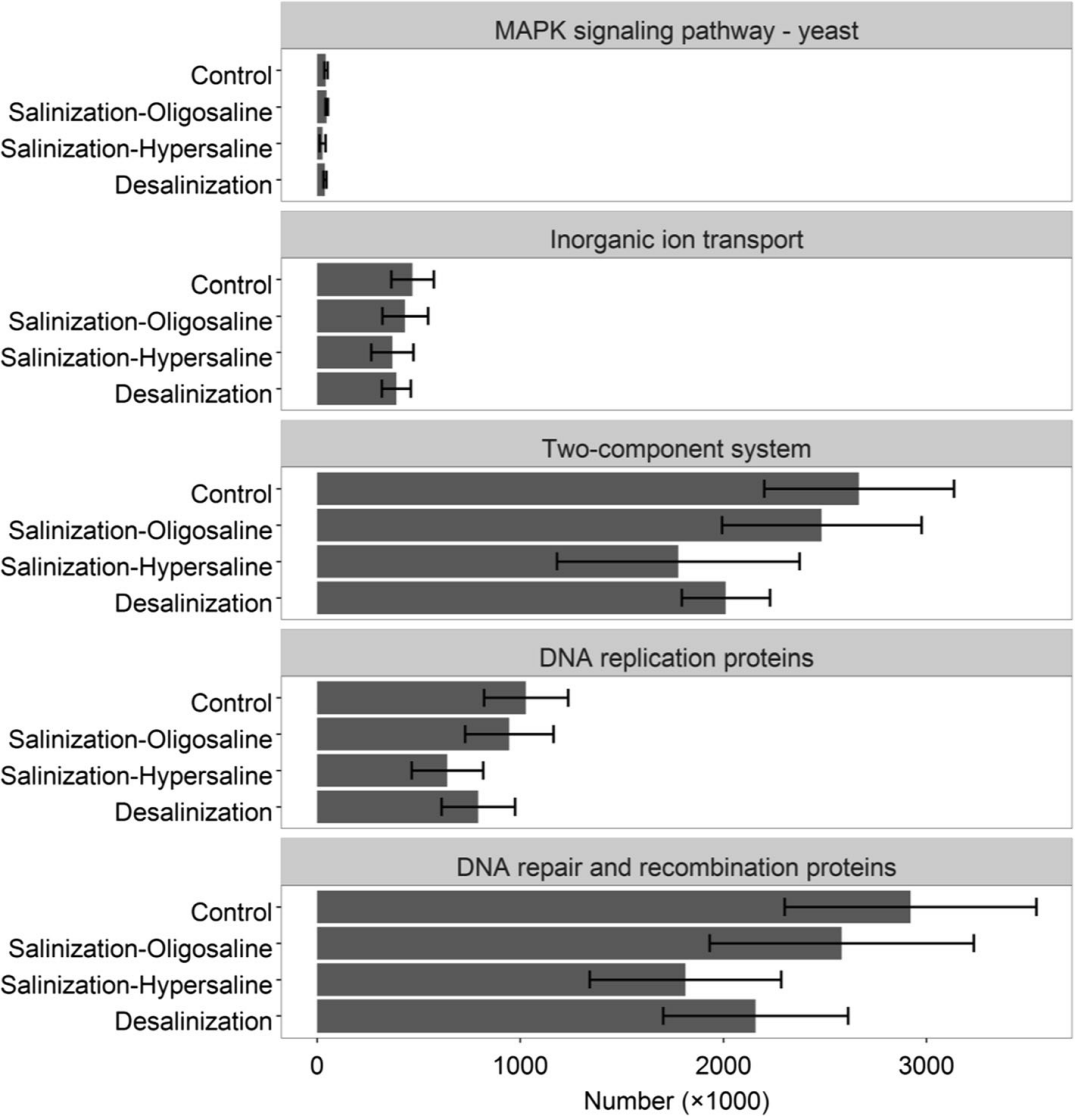
The schematic representation of the experimental design

The mesocosm experiment was conducted from 15 Jul. to 1 Dec. 2015 by self-made glass (Fig. 6). Six glass containers (each with a volume of 120 L) were divided into two groups (control and treatment) with three replicates in each group. The experimental water was taken from Lake Bosten, the largest inland freshwater lake in China (salinity: 0.03‰). Before being transferred into each container, the original water was stored in a single tank in a sterile room for 15 days to homogenize and stabilize the bacterial communities [26, 31]. Subsequently, each glass container was simultaneously filled with 100 L of original water. For the control group, three containers were placed aside without any disturbance for the course of the entire experimental period. The treatment group was designed to experience the extreme salinization-up process, followed by a desalinization-down process. To simulate the salinization, sterile crystallized salt was stepwise added at 15-day intervals to obtain an increasing salinity gradient of 1‰, 3‰, 10‰, 35‰, and 90‰. To simulate the desalinization, sterilized (120 °C for 30 min) original water was stepwise added to create a symmetric decreasing salinity gradient of 35‰, 10‰, 3‰, 1‰, and 0.03‰ at 15-day intervals. During the experiment, all incubations were carried out at 26 °C with caps covering the obtainers in order to prevent airborne bacteria from contaminating the system.

During experimental sampling, all tools were washed by sterile deionized water five times. An initial sample of 600 mL of water was taken before adding the sterile salt or additional water. For 16S rRNA genetic analysis, a subsample (400 mL) of water was immediately filtered on 0.22 μm pore size polycarbonate filters by a hand-driven vacuum pump. The filters were stored at − 80 °C before extraction of nucleic acids. The remaining water (200 mL) was transported to the laboratory for chemical analysis. Chemical analyses including total nitrogen (TN), total phosphorus (TP), and dissolved organic carbon (DOC), were performed according to standard methods [65]. During sampling, the physical properties including dissolved oxygen, salinity, temperature, and pH were determined by a multiparameter water quality sonde (YSI 6600 V2, USA).

### DNA extraction, PCR amplification, and sequencing

Total DNA was extracted using a FastDNA spin kit for soil (MP Bio-medical, Carlsbad, CA) according to the manufacturer’s instructions [66]. To amplify the V5-V6 hypervariable regions of 16S rRNA genes, the universal primers 789F (5′- TAGATACCCSSGTAGTCC-3′) and 1068R (5’-CTGACGRCRGCCATGC -3′) were used. The polymerase chain reaction (PCR) was performed in 50 μL reaction mixture containing 5 μL of 10 × PCR buffer, 4 μL of MgCl_2_ (25 mM), 3 μL of deoxynucleotide triphosphates (dNTPs, 2.5 mM each), 1 μL of each primer (10 μM), 30 ng template DNA, and 0.3 μL of *Taq* polymerase (5 UμL-1 Fermentas). PCR cycling was carried out in a thermocycler (Applied Biosystems Veriti Thermal Cycler) using a touchdown program: denaturation at 94 °C for 5 min, 11 cycles of denaturation at 94 °C for 1 min, annealing at 65 °C for 1 min, and extension at 72 °C for 1 min. Nineteen additional cycles were carried out at an annealing temperature of 55 °C, followed by a final extension at 72 °C for 10 min.

The pair-end sequencing was performed on an Illumina Miseq platform. Unique barcodes were added to each sample. The paired reads from each sample were initially merged with a minimum overlap of 100 and 8 maximum mismatches allowed in the overlap region. Primers and barcodes were trimmed so that the average Phred quality score for each read was above 20. After trimming, these reads were assembled by FLASH (https://github.com/dstreett/FLASH2). Only those reads with consecutive and identical bases, and without ambiguous bases, were used for further analysis. Chimera sequences were identified and removed using UCHIME [67]. The software QIIME was used to cluster the high-quality sequences into operational taxonomic units (OTUs) with a 0.03 cut-off value (equivalent to 97% similarity) [68]. The longest sequence in each cluster was chosen to be the representative sequence, which was annotated according to the SILVA database.

### Predictive metagenome analysis

We used the Phylogenetic Investigation of Communities by Reconstruction of Unobserved States (PICRUSt) software package (version 1.0.0, http://picrust.github.io/picrust/) to predict the metagenome functional content from the 16S rRNA dataset [57]. PICRUSt-compatible OTU tables were constructed using the closed-reference OTU picking protocol in QIIME against Greengenes by using the function ‘pick_reference_otus_through_otu_table.py’. The nearest sequenced taxon index was developed as a measure to describe the novelty of bacteria within an OTU table, with respect to previous sequenced genomes. The obtained OTU table was normalized to reflect their true abundance, and then predict_metagemones.py with default settings was applied to gain the predicted metagenomics table with Kyoto Encyclopedia of Genes and Genomes (KEGG) hierarchy collapse at level 3.

### Statistical analysis

All statistical analyses and visualization were carried out using *vegan*, *betapart*, and *ggplot2* packages in the R environment (version 3.2.2, http://www.r-project.org). Before alpha diversity analysis, the 16S rRNA data from individual sample were rarefied to 69,899 reads (the minimal read number across all samples). Faith’s phylogenetic diversity (PD) [69] and rarefied richness were calculated in QIIME. Shannon diversity was determined by *diversity* function. Pielou’s Evenness, *J*, was calculated based on Shannon diversity, H (*J* = H/log(richness)). The two-sided *t*-test was applied to determine if the *α*-diversity in different groups were significantly different from each other.

Prior to beta diversity analysis, all OTU abundance data were Hellinger-transformated [70]. Non-metric multidimensional scaling analysis (NMDS) was conducted with Bray-Curtis distance as well as (un)weighted UniFrac distance through the *metaMDS* function. The Bray-Curtis distance matrix was calculated by the *vegdist* function, and the (un)weighted UniFrac distance was calculated through QIIME. In order to access the variation of bacterial community, Sørensen dissimilarity matrix was determined using *beta.multi* function [71]. Additionally, we also performed a nested permutational multivariate analysis of variance (PERMANOVA, [72]) based on Bray-Curtis similarity using *adonis* function to test if the bacterial community composition in distinct groups were significantly different from each other.

Taxonomic and trait compositions were first assessed by computing Bray-Curtis distance between consecutive sampling dates across the entire duration of the study in order to detect major changes in community composition (i.e. more dissimilar values corresponding to higher distances).

## Additional files

Additional file 1: **Table S1.** The environmental properties of control and treatment groups. (DOCX 17 kb)

Additional file 2: **Figure S1.** The rarefaction plot indicating community observed OTUs based on 16S rRNA gene sequences. C1-C11 presents the control groups; S1-S6 presents the salinization groups, in which the salinity is 0.03‰, 1‰, 3‰, 10‰, 35‰, and 90‰, respectively; D1-D5 presents the desalinization groups, in which the salinity is 35‰, 10‰, 3‰, 1‰, and 0.03‰, respectively. (DOCX 306 kb)

Additional file 3: **Figure S2.** Non-metric multidimensional scaling ordination of bacterial communities in salinization-desalinization cycle and control groups based on unweighted and weighted Unifrac distance (DOCX 113 kb)

Additional file 4: **Figure S3.** Phylum-level changes of bacterial community composition during the control group (DOCX 224 kb)

Additional file 5: **Table S2.** The value of the nearest sequenced taxon index (NSTI) for each sample calculated by PICRUSt (DOCX 16 kb)

## Abbreviations

DOC: Dissolved organic carbon
KEGG: Kyoto Encyclopedia of Genes and Genomes
NMDS: Non-metric multidimensional scaling analysis
NSTI: Nearest sequenced taxon index
OTU: Operational Taxonomic Units
PCR: Polymerase chain reaction
PD: Faith’s phylogenetic diversity
PERMANOVA: Permutational Multivariate Analysis of Variance
PICRUSt: Phylogenetic Investigation of Communities by Reconstruction of Unobserved States
TN: Total nitrogen
TP: Total phosphorus
