# Supplementary material for: Low recovery of bacterial community after an extreme salinization-desalinization cycle
Source: BMC Microbiol. 2018 Nov 23;18:195. doi: 10.1186/s12866-018-1333-2 (PMC6251166; doi:10.1186/s12866-018-1333-2)
Supplement: Supplementary file 1 — Table S1. The environmental properties of control and treatment groups. (DOCX 17 kb) [file 12866_2018_1333_MOESM1_ESM.docx]

| Group | Salinity (‰) | DO (mg/L) | pH | DOC (mg/L) | TN (mg/L) | TP (mg/L) |
| --- | --- | --- | --- | --- | --- | --- |
| Control | 0.03 | 5.51 | 8.55 | 5.77 | 0.35 | 0.01 |
| Control | 0.03 | 6.44 | 8.74 | 5.56 | 0.32 | 0.01 |
| Control | 0.03 | 6.49 | 8.75 | 5.62 | 0.33 | 0.02 |
| Control | 0.03 | 5.76 | 8.71 | 5.67 | 0.34 | 0.02 |
| Control | 0.03 | 5.90 | 8.81 | 5.51 | 0.31 | 0.02 |
| Control | 0.03 | 6.19 | 8.87 | 5.74 | 0.32 | 0.02 |
| Control | 0.03 | 5.68 | 8.89 | 5.61 | 0.35 | 0.02 |
| Control | 0.03 | 5.91 | 8.77 | 5.55 | 0.31 | 0.01 |
| Control | 0.03 | 5.58 | 8.79 | 5.75 | 0.31 | 0.01 |
| Control | 0.03 | 5.98 | 8.77 | 5.61 | 0.35 | 0.01 |
| Control | 0.03 | 5.60 | 8.85 | 5.53 | 0.32 | 0.02 |
| Salinization | 0.03 | 5.56 | 8.51 | 5.60 | 0.31 | 0.02 |
| Salinization | 1.02 | 5.62 | 8.63 | 5.59 | 0.34 | 0.01 |
| Salinization | 2.97 | 5.95 | 8.73 | 5.78 | 0.34 | 0.02 |
| Salinization | 10.10 | 6.46 | 8.62 | 5.56 | 0.33 | 0.02 |
| Salinization | 34.94 | 6.29 | 8.78 | 5.80 | 0.36 | 0.02 |
| Salinization | 89.90 | 5.93 | 8.90 | 5.66 | 0.35 | 0.01 |
| Desalinization | 35.05 | 6.64 | 8.80 | 5.72 | 0.29 | 0.01 |
| Desalinization | 10.30 | 5.63 | 8.77 | 5.79 | 0.30 | 0.02 |
| Desalinization | 3.10 | 6.04 | 8.77 | 5.80 | 0.28 | 0.02 |
| Desalinization | 1.07 | 6.19 | 8.74 | 5.93 | 0.26 | 0.02 |
| Desalinization | 0.03 | 5.57 | 8.79 | 5.86 | 0.25 | 0.02 |
