# Supplementary figures and images for: Low recovery of bacterial community after an extreme salinization-desalinization cycle
Source: BMC Microbiol. 2018 Nov 23;18:195. doi: 10.1186/s12866-018-1333-2 (PMC6251166; doi:10.1186/s12866-018-1333-2)

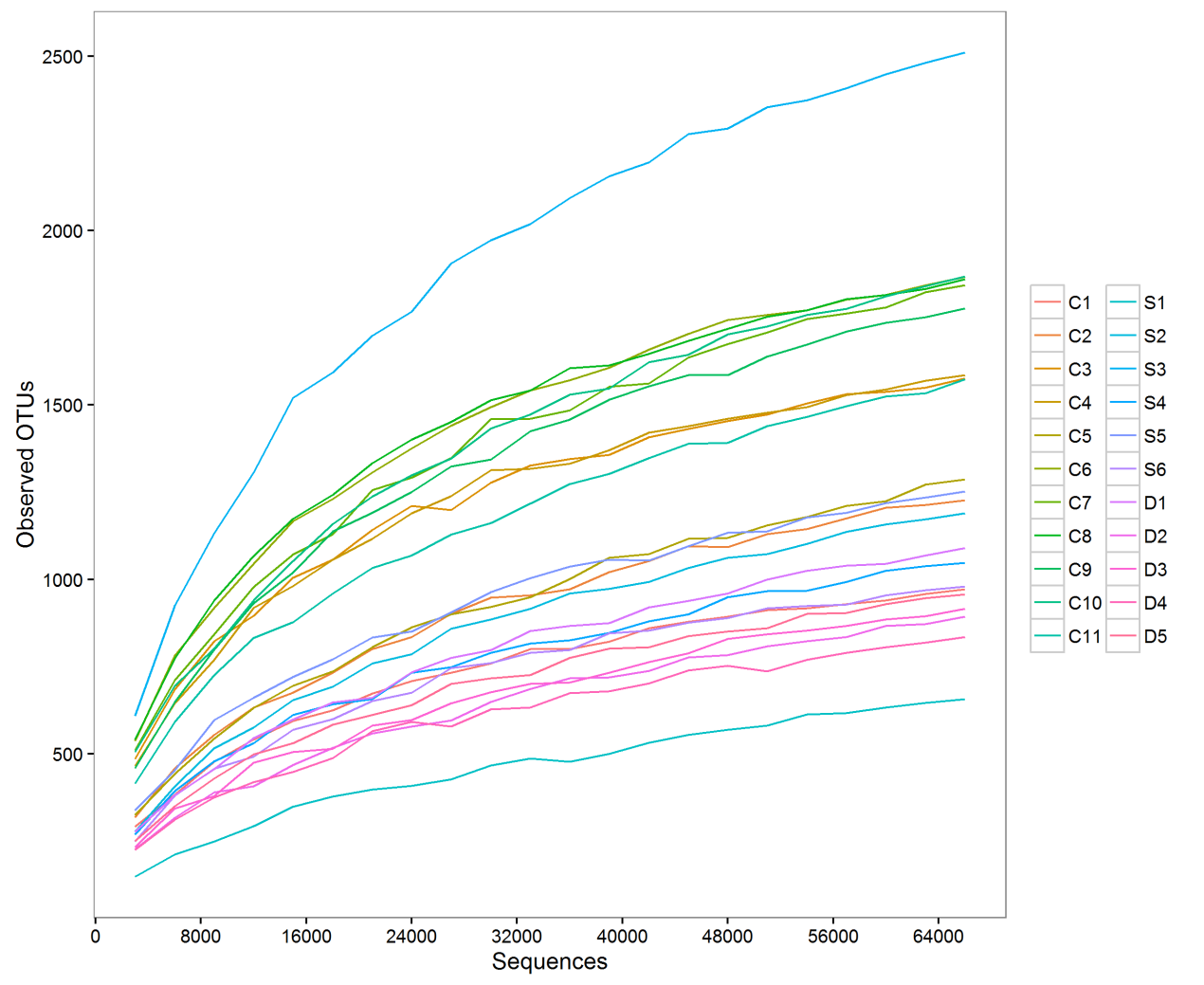

Supplement: Supplementary file 2 — Figure S1. The rarefaction plot indicating community observed OTUs based on 16S rRNA gene sequences. C1-C11 presents the control groups; S1-S6 presents the salinization groups, in which the salinity is 0.03‰, 1‰, 3‰, 10‰, 35‰, and 90‰, respectively; D1-D5 presents the desalinization groups, in which the salinity is 35‰, 10‰, 3‰, 1‰, and 0.03‰, respectively. (DOCX 306 kb) [file 12866_2018_1333_MOESM2_ESM.docx]

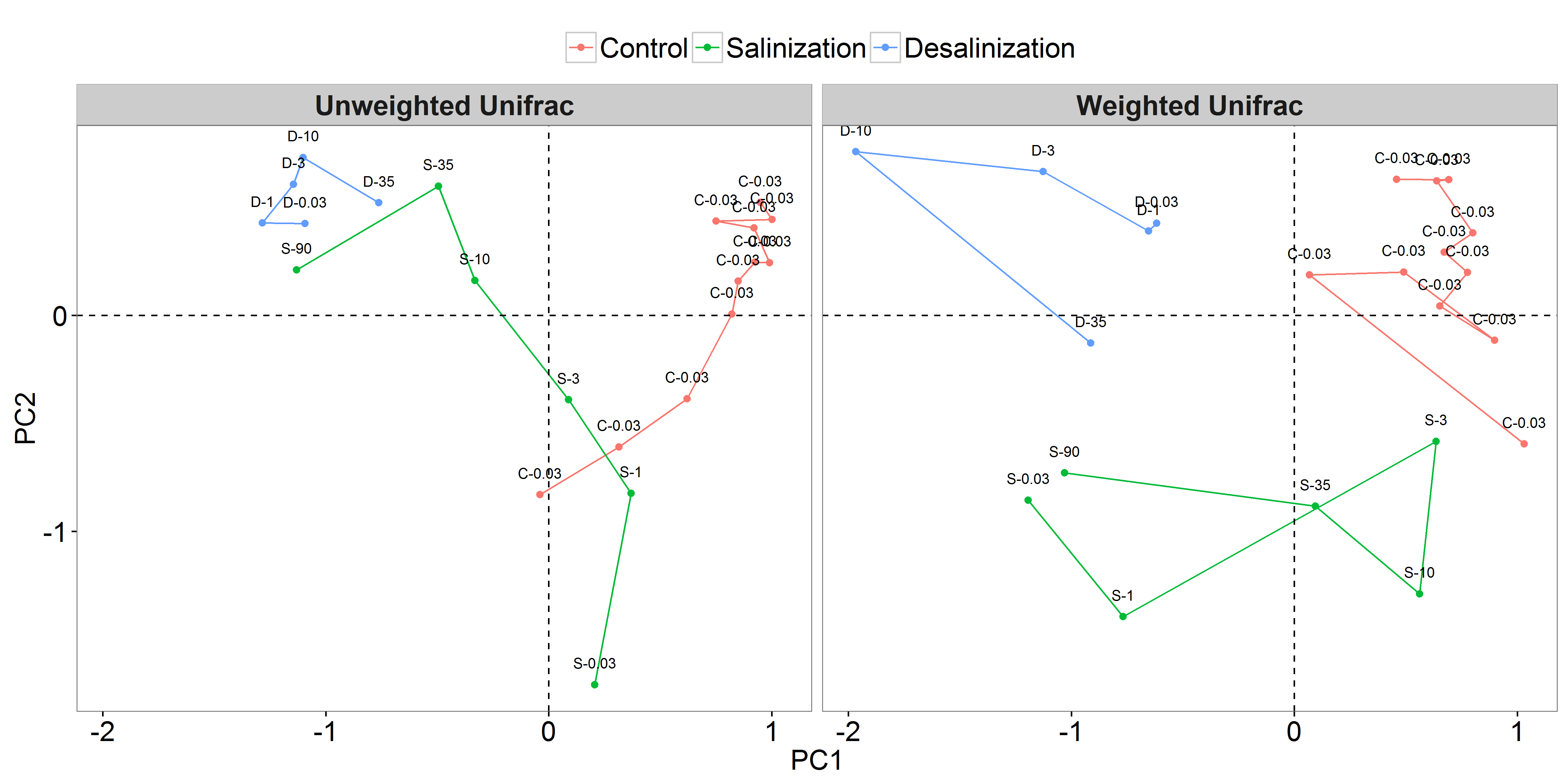

Supplement: Supplementary file 3 — Figure S2. Non-metric multidimensional scaling ordination of bacterial communities in salinization-desalinization cycle and control groups based on unweighted and weighted Unifrac distance (DOCX 113 kb) [file 12866_2018_1333_MOESM3_ESM.docx]

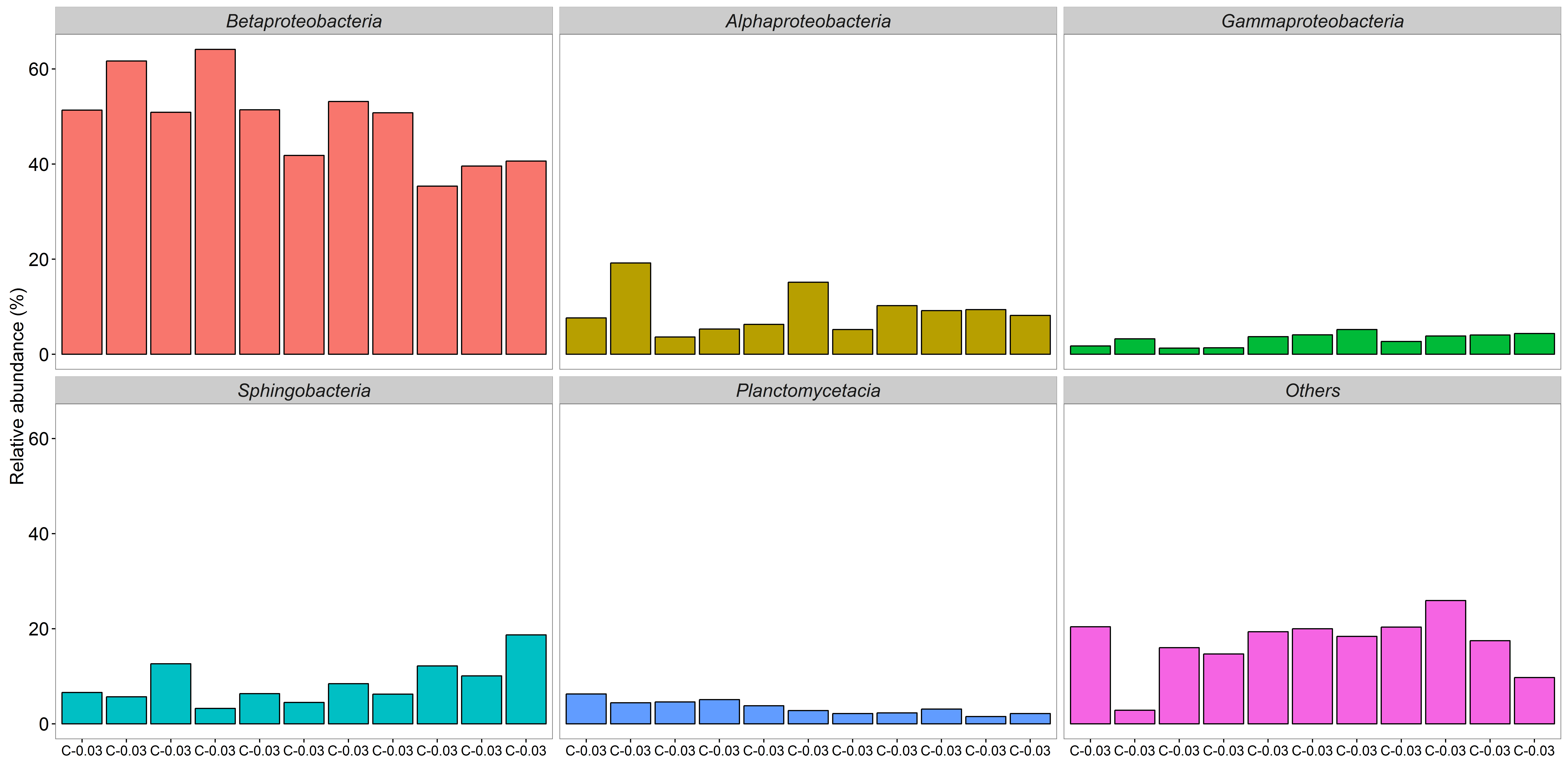

Supplement: Supplementary file 4 — Figure S3. Phylum-level changes of bacterial community composition during the control group (DOCX 224 kb) [file 12866_2018_1333_MOESM4_ESM.docx]
