# Supplementary material for: Low recovery of bacterial community after an extreme salinization-desalinization cycle
Source: BMC Microbiol. 2018 Nov 23;18:195. doi: 10.1186/s12866-018-1333-2 (PMC6251166; doi:10.1186/s12866-018-1333-2)
Supplement: Supplementary file 5 — Table S2. The value of the nearest sequenced taxon index (NSTI) for each sample calculated by PICRUSt (DOCX 16 kb) [file 12866_2018_1333_MOESM5_ESM.docx]

|  | group | Sampling  day | Weighted  NSTI value |
| --- | --- | --- | --- |
| C-0.03 | Control | 0 | 0.092 |
| C-0.03 |  | 15 | 0.106 |
| C-0.03 |  | 30 | 0.115 |
| C-0.03 |  | 45 | 0.134 |
| C-0.03 |  | 60 | 0.119 |
| C-0.03 |  | 75 | 0.131 |
| C-0.03 |  | 90 | 0.134 |
| C-0.03 |  | 105 | 0.131 |
| C-0.03 |  | 120 | 0.146 |
| C-0.03 |  | 135 | 0.171 |
| C-0.03 |  | 150 | 0.132 |
| S-0.03 | Oligosaline condition | 0 | 0.047 |
| S-1 |  | 15 | 0.086 |
| S-3 |  | 30 | 0.143 |
| S-10 | Hypersaline condition | 45 | 0.178 |
| S-35 |  | 60 | 0.226 |
| S-90 |  | 75 | 0.067 |
| D-35 | Desalinization | 90 | 0.095 |
| D-10 |  | 105 | 0.134 |
| D-3 |  | 120 | 0.136 |
| D-1 |  | 135 | 0.131 |
| D-0.03 |  | 150 | 0.131 |
